# Supplementary material for: Cryptomphalus aspersa Egg Extract Protects against Human Stem Cell Stress-Induced Premature Senescence
Source: Int J Mol Sci. 2024 Mar 27;25(7):3715. doi: 10.3390/ijms25073715 (PMC11011511; doi:10.3390/ijms25073715)
Supplement: Supplementary file 1 [file ijms-25-03715-s001.zip › ijms-2837671-supplementary.pdf]

Supplementary file

A

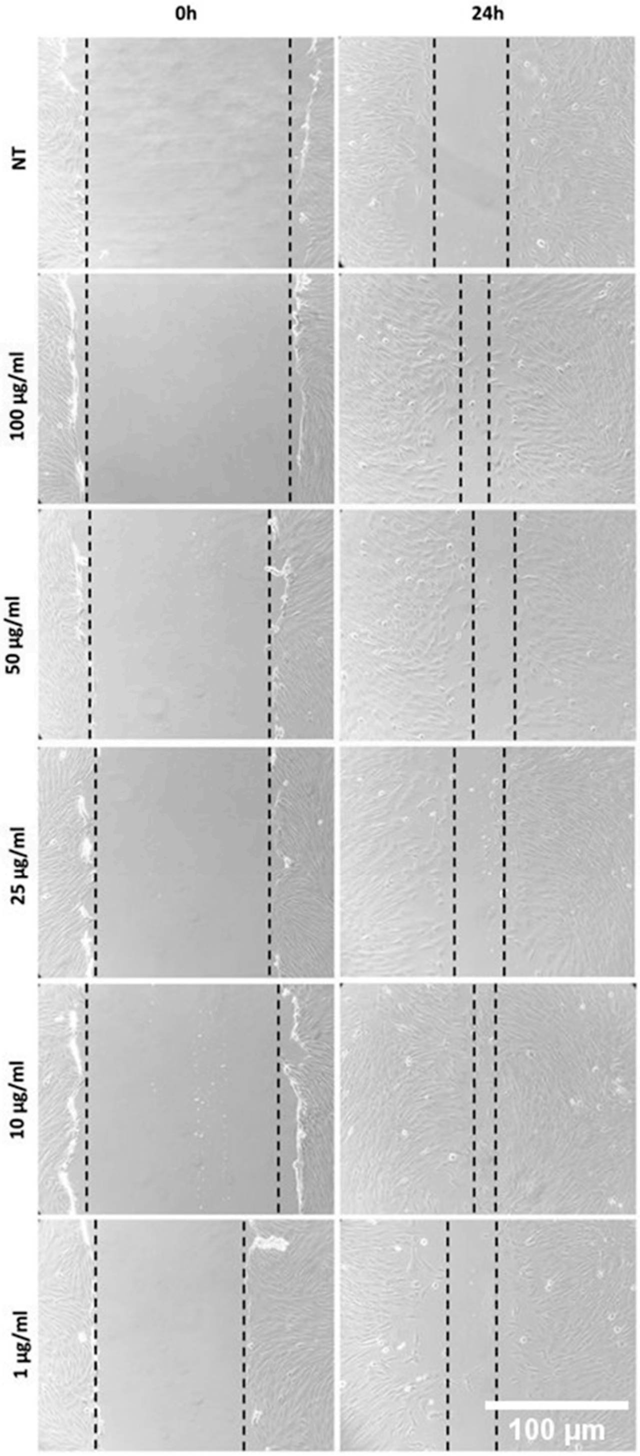

B

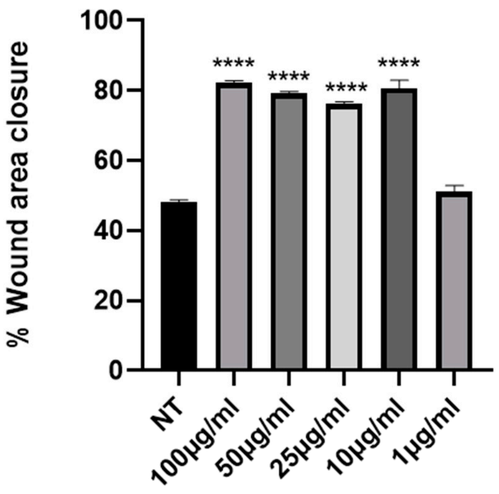

Supplementary figure

Supplementary Figure S1 legend: ***Cryptomphalus aspersa*** **egg extract (CAEE) exhibits potent wound healing ability.** (A) Representative images of “wounds” treated with various concentrations (1μg/ml-100μg/ml) of the egg extract (CAEE) or “wounds” that have not been treated with the extract (NT), immediately after “wound” infliction (0h) and 24h post “wound” infliction (24h). (B) Graph demonstrating “would healing” expressed as the percentage of original “wound” area closure after 24h, for “wounds” treated with various concentrations of the extract (CAEE) and “wounds” not treated with the extract (NT). Values shown are the means ± S.E from three different experiments with WJ-MSCs derived from three different donors (n=3) treated (CAEE) or untreated (NT). Asterisk marks statistical significance (**** = $p < 0.0001$).
